# Preparation of New *Sargassum fusiforme* Polysaccharide Long-Chain Alkyl Group Nanomicelles and Their Antiviral Properties against ALV-J

**DOI:** 10.3390/molecules26113265

**Published:** 2021-05-28

**Authors:** Yuhao Sun, Xiaolin Chen, Hong Liu, Song Liu, Huahua Yu, Xueqin Wang, Yukun Qin, Pengcheng Li

**Affiliations:** 1 CAS and Shandong Province Key Laboratory of Experimental Marine Biology, Center for Ocean Mega-Science, Institute of Oceanology, Chinese Academy of Sciences, Qingdao 266071, China; yhsun@qnlm.ac (Y.S.); liuhong215@mails.ucas.edu.cn (H.L.); sliu@qdio.ac.cn (S.L.); yuhuahua@qdio.ac.cn (H.Y.); xueqinwang@qdio.ac.cn (X.W.); ykqin@qdio.ac.cn (Y.Q.); 2 Laboratory for Marine Drugs and Bioproducts, Pilot National Laboratory for Marine Science and Technology (Qingdao), No. 1 Wenhai Road, Qingdao 266237, China

**Keywords:** *Sargassum fusiforme*, polysaccharide, long-chain alkyl group, antiviral activity, ALV-J

## Abstract

Avian leukosis virus subgroup J (ALV-J) is an immunosuppressive virus which has caused heavy losses to the poultry breeding industry. Currently, there is no effective medicine to treat this virus. In our previous experiments, the low-molecular-weight *Sargassum fusiforme* polysaccharide (SFP) was proven to possess antiviral activity against ALV-J, but its function was limited to the virus adsorption stage. In order to improve the antiviral activity of the SFP, in this study, three new SFP long-chain alkyl group nanomicelles (SFP-C12M, SFP-C14M and SFP-C16M) were prepared. The nanomicelles were characterized according to their physical and chemical properties. The nanomicelles were characterized by particle size, zeta potential, polydispersity index, critical micelle concentration and morphology. The results showed the particle sizes of the three nanomicelles were all approximately 200 nm and SFP-C14M and SFP-C16M were more stable than SFP-C12M. The newly prepared nanomicelles exhibited a better anti-ALV-J activity than the SFP, with SFP-C16M exhibiting the best antiviral effects in both the virus adsorption stage and the replication stage. The results of the giant unilamellar vesicle exposure experiment demonstrated that the new virucidal effect of the nanomicelles might be caused by damage to the phospholipid membrane of ALV-J. This study provides a potential idea for ALV-J prevention and development of other antiviral drugs.

## 1. Introduction

*Sargassum fusiforme*, perennial warm-temperature algae, belong to the family Sargassaceae, phylum Ochrophyta, class Phaeophyceae, which is widely distributed in the eastern coastal areas of Asia [1]. In China and South Korea, *S. fusiforme* has been used as food and traditional medicine for a long time [2,3]. As an important raw material of alginate, *S. fusiforme* also possesses a very high commercial value [4]. Many bioactive components from *S. fusiforme* such as proteins, sterols and especially polysaccharides have been investigated. In recent decades, the *S. fusiforme* polysaccharide (SFP) has attracted much attention from researchers because of its various biological activities. Studies have proven that the SFP has antioxidant [5], immunoregulatory [6,7], antitumor [8], antiaging [9], hypoglycemic and other effects [10]. Our previous studies found that the SFP with a molecular weight of 9 kDa could inhibit avian leukosis virus subgroup J (ALV-J) in vitro. It was shown to prevent ALV-J from adsorbing onto host cells. In addition, the SFP was discovered to relieve immune suppression, growth inhibition and organ damage observed in chickens caused by ALV-J in vivo [11]. However, how to increase the antiviral activity of the SFP so that it is not limited to the adsorption stage will be a significant problem in utilizing the SFP to develop antiviral drugs.

In the last few years, nanoparticles have been used as important diagnostic and therapeutic tools in medicine [12,13,14,15]. Nanomicelles, a kind of nanoparticles, have been widely investigated because of their easy preparation, great biocompatibility and high drug loading efficiency [16]. Traditional nanomicelles are composed of amphiphilic molecules which feature two completely different regions that exhibit opposite affinities for water, and loaded drugs. Amphiphilic molecules can form a special internal hydrophobic and external hydrophilic colloidal structure at appropriate temperatures and concentrations [17]. Due to their amphiphilic properties, nanomicelles can be used as carriers to deliver lipophilic drugs contained within the hydrophobic core, which greatly improves the solubility and stability of drugs, thus improving the permeability of drugs into the intestines [18,19]. At present, nanomicelle studies mainly focus on drug delivery systems, such as alginate [20,21,22,23], chitosan [24,25,26] and heparin [27,28]. However, some studies have shown that drugs with direct hydrophilic or hydrophobic modifications and self-assembly into nanomicelles might improve the pharmaceutical effects. Buchy et al. [29] reported that squalene conjugates of sunitinib could self-assemble into nanomicelles, and these conjugates exhibited stronger inhibition in HUVECs (human umbilical vein endothelial cells) which were involved in the tumor vessel formation with an IC_50_ of 4.1 ± 0.2. Mei et al. [27] developed doxorubicin–heparin micelles, and these nanoparticles exhibited a more efficient antitumor activity along with antiangiogenic and antimetastatic effects. Compared with traditional nanomicelles, the direct drug modification method could reduce the introduction of hydrophobic or hydrophilic fragments from the carrier, which has no direct therapeutic effects and might bring side effects.

Therefore, this paper aimed to prepare several SFP nanomicelles by direct hydrophobic modification of the SFP. The morphology and physiochemical properties of the micelles were characterized. Then, the anti-ALV-J activity of the micelles was determined, and the antiviral mechanism was preliminarily analyzed.

## 2. Materials and Methods

### 2.1. Algae and Reagents

*S. fusiforme* was purchased from Rongcheng Xufeng Aquatic Products Co., Ltd. (Weihai, China). Standard sugars (glucose, rhamnose, xylose, mannose, galactose, glucuronic acid, fucose and dextran with molecular weights of 1000, 5000, 25,000, 50,000, 80,000, 270,000 and 510,000, respectively) and 1,2-dipalmitoyl-3-trimethylammonium-propane (DOTAP) were purchased from Sigma Chemicals Co. (Shanghai, China). Lauroyl chloride, myristoyl chloride and palmitoyl chloride were produced by Shanghai Macklin Biochemical Technology Co., Ltd. (Shanghai, China). An Avian Leukosis Virus p27 Antigen test kit was purchased from IDEXX Laboratories Inc. (Beijing, China). The manufacturer of 1,2-dioleoyl-sn-glycero-3-phosphocholine (DOPC) and 1,2-dioleoyl-sn-glycero-3-[phospho-rac-(1-glycerol)] (DOPG) was Shanghai Aladdin Biochemical Technology Co., Ltd. (Shanghai, China). The DF-1 cell line was obtained from the Institutes for Biological Sciences Cell Bank, Chinese Academy of Sciences (Shanghai, China). ALV-J strain NX0101 was kindly gifted by Prof. Cheng, Shandong Agricultural University (Taian, China).

### 2.2. Preparation of the SFP

*S. fusiforme* was washed and dried to constant weight at 50 °C and then crushed. The algae powder was mixed with 95% ethanol with a solid/liquid ratio of 1:10 at 80 °C for 2 h. The mixture was filtered, and the residue was refluxed again to remove the lipids and pigments. After filtration and drying, 100 g algae powder was extracted with 3 L distilled water at 80 °C for 4 h. The mixture was filtered through gauze, and CaCl_2_ was added to a concentration of 0.2 mol/L. The residue was separated by centrifugation, and the solution was dialyzed against distilled water for 48 h. Subsequently, the solution was concentrated to approximately 500 mL, and the original polysaccharide was precipitated by adding 1.5 L of ethanol followed by lyophilization. Additionally, according to our previous methods [30], the prepared original polysaccharide was dissolved in distilled water to a concentration of 20 mg/mL, and then 30% hydrogen peroxide was added to a final concentration of 1.5%. HCl was used to adjust the pH value to 4. The polysaccharide was degraded at 90 °C for 90 min with stirring. After cooling to room temperature, the reaction solution was neutralized and dialyzed for 48 h. Finally, the SFP was yielded by lyophilization.

The FT-IR spectrum of the SFP was recorded using a Nicolet-360 FT-IR spectrometer (Thermofisher, Waltham, MA, USA). The weight average molecular weight (Mw) was measured by HPGPC [31]. The total sugar and sulfate contents were detected by the phenol–sulfuric acid method [32] and the barium chloride gelatin method [33], respectively. The 1-phenyl-3-methyl-5-pyrazolone (PMP) precolumn derivation HPLC method [34] was used to determine the monosaccharide composition of the SFP. The ^1^H-NMR was measured by a JEOL JNM-ECP 600 spectrometer (Tokyo, Japan) using dimethyl sulfoxide-d6 (DMSO-d6) as a solvent.

### 2.3. Preparation and Characterization of SFP Long-Chain Alkyl Group Derivatives

The SFP (0.5 g) and 4-dimethylaminopyridine (0.83 g) were dissolved in 30 mL of formamide. Then, the corresponding fatty acyl chloride (1.38 mL of lauroyl chloride, 1.58 mL of myristoyl chloride or 1.67 mL of palmitoyl chloride) was added dropwise under nitrogen protection and reacted for 12 h at 90 °C. The mixture was precipitated with three equivalents of anhydrous ethanol and then centrifuged. The precipitate was washed twice with anhydrous ethanol and dissolved in water. The solution was dialyzed against distilled water using a 500–1000-Da Mw cutoff dialysis tube for 48 h. The SFP long-chain alkyl group derivatives were obtained after lyophilization. The yield (%) was calculated using Equation (1):

(1) $$\mathrm{Yield}\;\left(\%\right)=\frac{\mathrm{derivative}\;\mathrm{weight}\;}{\mathrm{SFP}\;\mathrm{weight}\;+\;\mathrm{fatty}\;\mathrm{acyl}\;\mathrm{chloride}\;\mathrm{weight}}$$

The FT-IR and ^1^H-NMR spectra of the SFP long-chain alkyl group derivatives were measured, and the determinations of the total sugar and sulfate contents were the same as the methods described in Section 2.2.

### 2.4. Preparation of SFP Nanomicelles

The dialysis method was used to prepare SFP nanomicelles as follows. Ten milligrams of SFP long-chain alkyl group derivatives were dissolved in 10 mL formamide. The solution was dialyzed against distilled water using a dialysis tube with a 500–1000-Da Mw cutoff for 48 h. For the first 12 h, the water was changed every 2 h. After dialyzing, the solution was concentrated and lyophilized for further use.

### 2.5. Characterization of SFP Nanomicelles

#### 2.5.1. Determination of Particle Size, Zeta Potential and Polydispersity Index

The micelles were dissolved in water at a concentration of 1 mg/mL. The particle sizes, zeta potentials and PDIs were measured by dynamic light scattering using a Zetasizer ZS90 (Malvern, UK).

#### 2.5.2. Detection of the Critical Micelle Concentration

The CMC was estimated by the pyrene fluorescence probe method [35]. Briefly, a 1 mg/mL micelle solution was prepared and diluted with distilled water to 0.25 mg/mL, 0.1 mg/mL, 0.05 mg/mL, 0.025 mg/mL, 0.005 mg/mL, 0.001 mg/mL, 0.00025 mg/mL and 0.00005 mg/mL. A pyrene/acetone solution with a concentration of 6 × 10^−4^ mg/mL was precisely prepared, and 30 μL of the solution were put into a 5-mL centrifuge tube and kept in a 40 °C water bath in the dark for 30 min to completely volatilize the acetone. Subsequently, 3 mL of the indicated micelle solution were added and the mixture was maintained in a 40 °C water bath in the dark with oscillation overnight. A fluorescence spectrophotometer was used to determine the fluorescence intensity of the micelle solution. The excitation wavelength was set to 339 nm, the scanning wavelength was set to 350–500 nm, and the scanning speed was 240 nm/min. The peaks at 372 nm (I1) and 383 nm (I3) from the emission spectrum were recorded, and the CMC was calculated by plotting I1/I3 as the ordinate and the logarithmic micelle concentration as the abscissa.

#### 2.5.3. Observation of Micelle Morphology

Scanning electron microscopy (SEM) was utilized to observe the SFP nanomicelle morphology. A droplet of each nanomicelle sample was dropped onto a glass slide and dried. Afterwards, the glass slide was coated with a thin layer of gold and observed by SEM (S-3400N, Hitachi, Tokyo, Japan).

### 2.6. Cytotoxicity Test

The cytotoxicities of the SFP derivatives and micelles were determined in DF-1 cells using the Cell Counting Kit-8 (CCK-8) assay. Briefly, DF-1 cells grew in 96-well plates to a monolayer and were then covered with 100 μL of the sample solution (the SFP long-chain alkyl group derivatives and SFP long-chain alkyl group derivative micelles dissolved in DMEM with 2% FBS) at different concentrations for 24 h. All of the treatments were performed in triplicate. The cells were washed with PBS three times, and 100 μL DMEM and 10 μL 2-(2-methoxy-4-nitrophenyl)-3-(4-nitrophenyl)-5-(2,4-disulfophenyl)-2H-tetrazolium sodium salt were added. The absorbance at 450 nm was measured 3 h later. The relative cell survival rate was calculated using Equation (2):

(2) $$\mathrm{Survival}\;\mathrm{rate}\;\left(\%\right)=\frac{\mathrm{sample}\;\mathrm{absorbance}\;-\;\mathrm{blank}\;\mathrm{control}\;\mathrm{absorbance}}{\mathrm{control}\;\mathrm{absorbance}\;-\;\mathrm{blank}\;\mathrm{control}\;\mathrm{absorbance}}$$

### 2.7. Anti-ALV-J Activity In Vitro

#### 2.7.1. Detection of the ALV p27 Antigen

DF-1 cells grew in 96-well plates to a monolayer, and then the cells were inoculated with 100 μL ALV-J and supplied with 100 μL of a 1 mg/mL sample solution (dissolved in DMEM with 2% FBS) for 1.5 h at 37 °C with three duplicates. Then, the mixture was washed away and the cells were covered with 100 μL of a corresponding 0.5 mg/mL sample solution. Meanwhile, the cell control and virus control were also set. After 48 h, the ALV p27 antigen in the supernatant was measured using an IDEXX ALV p27 antigen test kit according to the instructions. The relative ALV p27 antigen expression was calculated using Equation (3):

(3) $$s/p=\frac{\mathrm{sample}\;\mathrm{mean}\;-\;\mathrm{negative}\;\mathrm{control}\;\mathrm{mean}}{\mathrm{positive}\;\mathrm{control}\;\mathrm{mean}\;-\;\mathrm{negative}\;\mathrm{control}\;\mathrm{mean}}$$

The negative control and the positive control were given in the ELISA kit.

#### 2.7.2. Detection of the Relative ALV-J Gene Expression

DF-1 cells grew in a 12-well plate to a monolayer; then, they were inoculated with 200 μL ALV-J and supplied with 200 μL of a 0.4 mg/mL sample solution (dissolved in DMEM with 2% FBS) with three duplicates for 1.5 h at 37 °C. The supernatant was removed, and the cells were washed with PBS three times. After that, the DF-1 cells were recovered in 1 mL of the corresponding sample solution (dissolved in DMEM with 2% FBS) and maintained for 48 h. Finally, the cells were collected to extract total RNA with an EZNA Total RNA Kit I (OMEGA, Norcross, GA, USA), and reverse transcription and real-time PCR were performed using the PrimeScript^TM^ RT reagent Kit with gDNA Eraser and the SYBR^®^ Premix ExTaq^TM^ Kit (Takara BIO INC, Dalian, China), respectively. The ALV-J primers were designed according to our previous report [11].

### 2.8. Action Stage Assay

DF-1 cells grew in 96-well plates to a monolayer at 37 °C with 5% CO_2_ and were then supplied with SFP-C16 or SFP-C16M for different administrations.

Before adsorption (BA): First, 100 μL of 0.2 mg/mL SFP, SFP-C16 and SFP-C16M (dissolved in DMEM with 2% FBS) were added separately to DF-1 cell monolayers and incubated for 2 h. Then, the sample solution was removed, and the cells were infected with ALV-J for 1.5 h. After incubation, the unabsorbed ALV-J was washed away, and the DF-1 cells were covered with DMEM (containing 2% FBS) for 24 h.

Adsorption (Ad): The DF-1 monolayer was inoculated with 100 μL ALV-J and mixed with 100 μL of 0.4 mg/mL SFP, SFP-C16 or SFP-C16M (dissolved in DMEM with 2% FBS). After 1.5 h, the cells were washed with PBS and supplied with DMEM (containing 2% FBS) for 24 h.

After adsorption (AD): The DF-1 cells were inoculated with 100 μL ALV-J for 1.5 h. After washing, the cells were recovered with 100 μL of 0.2 mg/mL SFP, SFP-C16 or SFP-C16M (dissolved in DMEM with 2% FBS) and maintained for 24 h.

Ultimately, each supernatant was collected to detect the ALV p27 antigen using an IDEXX ALV p27 antigen test kit. The cell control and virus controls were also set. All of the treatments were performed in triplicate.

### 2.9. Giant Unilamellar Vesicle (GUV) Exposure Experiment

GUVs were prepared using the gentle hydration method [36]. DOPC, DOPG and DOTAP were dissolved in a chloroform–methanol solution (2:1 *v*/*v*) to a concentration of 18 mg/mL, 2 mg/mL and 2 mg/mL, respectively. Then, 50 μL of the DOPC solution were mixed with 50 μL of the DOPG or DOTAP solution in a 10-mL glass tube. The mixed phospholipid solution was rotationally evaporated under nitrogen until a homogeneous phospholipid film formed on the glass tube wall. The tube was then placed in a vacuum dryer for 6 h to ensure that all organic reagents volatilize. After that, approximately 4 mL of a 0.1 mol/L sucrose solution were slowly added to the test tube which was placed in an incubator at 37 °C and gently hydrated for 24 h to form GUVs. The GUVs were diluted with 0.1 mol/L glucose to observe the morphology of the GUVs under a microscope before the exposure experiment. The nanomicelles were dissolved in a 0.1 mol/L glucose solution, and the solution was used to dilute the GUVs to the indicated concentration. After mixing, the morphologies of the GUVs were observed under a microscope at 0 min, 10 min, 30 min, 90 min, 6 h and 24 h. The solution inside and outside the GUVs produced different refractive indices on the surface under the microscope and caused the GUVs to be visible in the bright field. The diameters of the prepared GUVs were about 10–100 μm.

## 3. Results and Discussion

### 3.1. Characterization of the SFP

**Figure 1:**
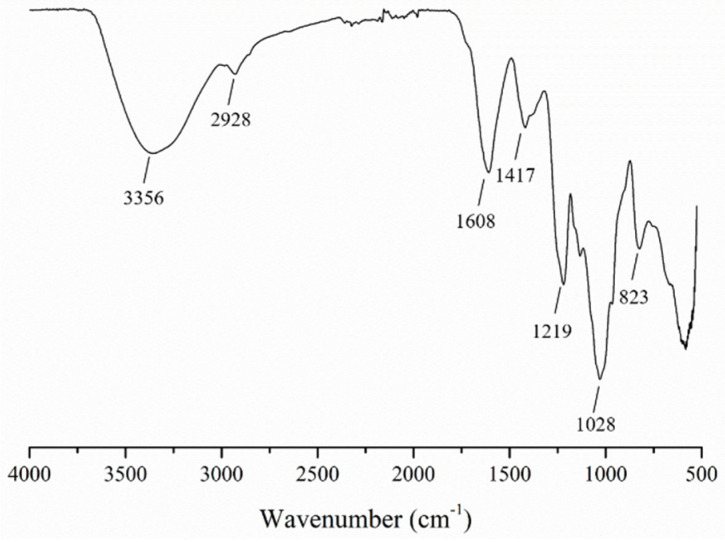
FT-IR spectrum of the SFP.

The SFP with a weight average molecular weight of 9 kDa (the polydispersity was 2.82) was obtained using hot water extraction and hydrogen peroxide oxidative degradation. The HPGPC (high performance gel permeation chromatography) profile of the SFP is displayed in Figure S1. In addition, the chemical composition is shown in Table 1. The results indicated that the total sugar content of the SFP was 54 ± 1% and the sulfate content was 37 ± 1%. The SFP was mainly composed of fucose, followed by galactose, a small amount of mannose, xylose, rhamnose and glucuronic acid and a relatively small percentage of glucose. Based on the previous studies [37,38,39,40], in Figure 1, the O–H and C–H stretching vibrations appeared at 3356 and 2928 cm^−1^, respectively, in the FT-IR spectrum. The absorption peaks at 1608 and 1417 cm^−1^ represented COO^–^ asymmetric and symmetric stretching vibrations, respectively. The peak at 1219 cm^−1^ was from the S=O stretching vibration. The absorption peaks at 1028 cm^−1^ and 823 cm^−1^ corresponded to the C–O–H deformation vibration and the C–O–S symmetrical stretching vibration, respectively. The FT-IR spectrum indicated that the SFP was a typical sulfated marine algae polysaccharide.

### 3.2. Characterization of SFP Long-Chain Alkyl Group Derivatives

**Figure 2:**
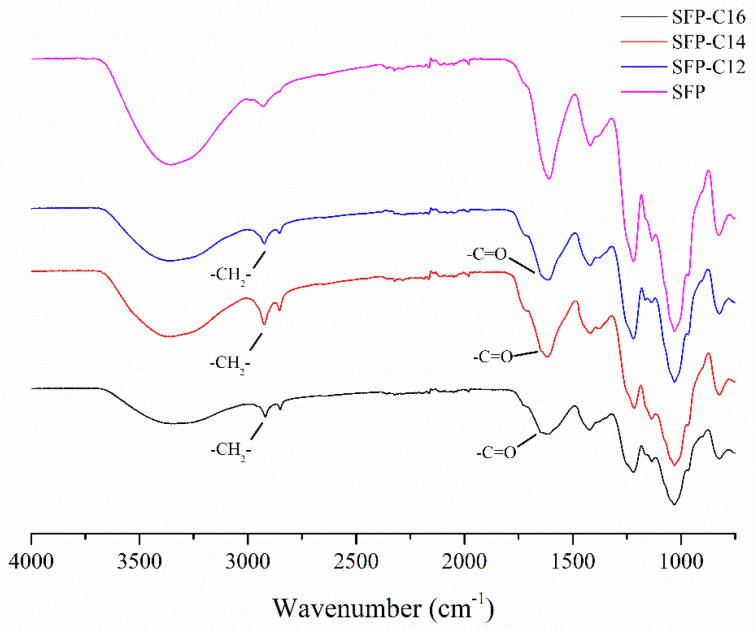
FT-IR spectra of SFP long-chain alkyl group derivatives.

As shown in Table 2, the yields of the three SFP products were approximately 9%, and the total sugar and sulfate contents were decreased compared with those of the SFP. This might be due to the successful grafting of long-chain alkyl groups. In Figure 2, in contrast to the SFP, except for the single absorption peak of C–H at 2928 cm^−1^, the double absorption peaks of SFP-C12, SFP-C14 and SFP-C16 appeared at approximately 2928 cm^−1^ and 2915 cm^−1^, respectively, which indicated the presence of absorption peaks from –CH2– in the long-chain alkyl group. Additionally, the original C=O peaks at 1620 cm^−1^ widened and tended to divide, which was mainly affected by the C=O in the long-chain fatty acyl chloride.

**Figure 3:**
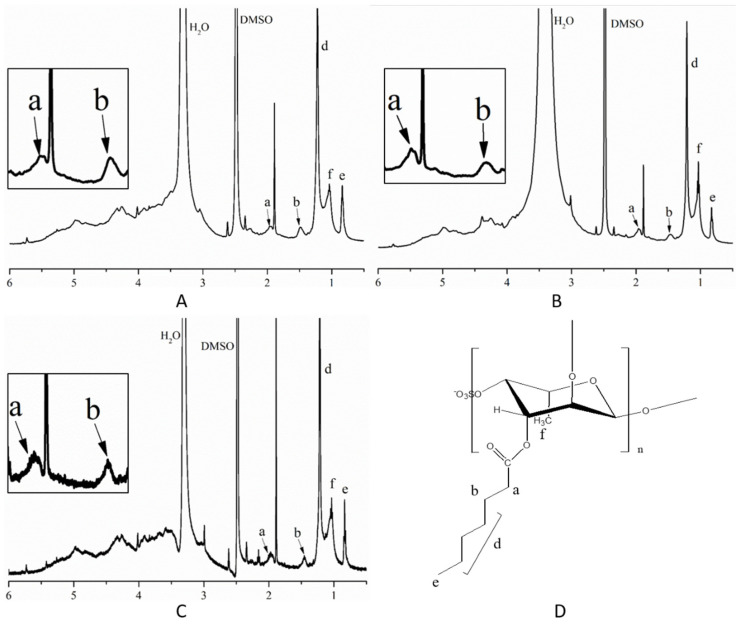
^1^H-NMR spectra of the SFP long-chain alkyl group derivatives (d = 9, 11, 13). (**A**) ^1^H-NMR spectra of SFP-C12; (**B**) ^1^H-NMR spectra of SFP-C14; (**C**) ^1^H-NMR spectra of C16; (**D**) The chemical structures of SFP long-chain alkyl group derivatives. The peaks a, b, c, d, e in (**A**–**C**) represent different proton absorption and they are indicated in (**D**).

The ^1^H-NMR spectra of the SFP derivatives are shown in Figure 3. According to previous results [41,42,43], the H1–H5 absorption peaks in the sugar ring appeared at 3.0–5.5 ppm. Peaks e (0.84 ppm) represented –CH_3_ at the end of the chain of the long-chain alkyl group. The –CH_3_ adsorption peaks of fucose were approximately 1.03 ppm (f). Peaks a (approximately 1.92 ppm) were related to the –CH_2_– proton linked with the –C=O group. The peaks at 1.49 ppm (b) were attributed to the –CH_2_– proton that was close to peak a in the long-chain alkyl group. Absorption peaks d (1.22 ppm) indicated the other –CH_2_– protons in the long-chain alkyl group excluding peaks b and a. The exact chemical shift of the indicated protons is shown in the Supplementary Materials, Figure S1. Combining the FT-IR and ^1^H-NMR results, the SFP derivatives were successfully prepared.

### 3.3. Characterization of SFP Nanomicelles

The particle sizes, polydispersity indexes (PDIs), zeta potentials and CMCs (critical micelle concentrations) of the SFP nanomicelles are shown in Table 3. The particle sizes of the three micelles were all approximately 200 nm, which was consistent with the particle size range of micelles. Additionally, the PDIs of the three kinds of micelles were low, indicating that the nanomicelles were uniform. Since SFP is a sulfate polysaccharide, SFP nanomicelles carry a negative charge. The charge level had a great influence on the particle stability. Zeta potential is a basic parameter that characterizes the surface charge of micelles [44]. If the absolute zeta potential value is low, the nanoparticles accumulate and become unstable. In contrast, the nanoparticles would repel each other, increasing the stability of the whole colloid. Amphiphilic oleic acid-modified carboxymethyl chitosan micelles with a zeta potential of 36.7 ± 2.64 mV [45] and hyaluronic acid conjugate micelles with a zeta potential of −35.5 mV [46] were both very stable. Our results showed that the zeta potentials of the three micelles were −39.37, −35.1 and −37.8 mV for SFP-12M, SFP-14M and SFP-16M, respectively, which indicated excellent stability. The CMC is the concentration of an amphiphilic molecule when it begins to self-assemble into micelles in an aqueous solution [47]. Low CMC of nanomicelles implies that they do not depolymerize easily in solution. The results of the pyrene fluorescence probe assay showed that the CMCs of the three micelles were 8.8, 5.0 and 12.1 μg/mL for SFP-12M, SFP-14M and SFP-16M, respectively, which also proved that the three micelles were very stable.

**Figure 4:**
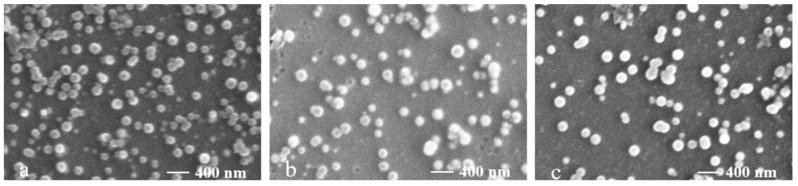
SEM spectra of the SFP nanomicelles. Scale bar: 400 nm. (**a**) SFP-C12M; (**b**) SFP-C14M; (**c**) SFP-C16M.

Furthermore, SEM was used to observe the morphologies of the nanomicelles. The results in Figure 4 demonstrate that the micelles were smooth, spherical, uniform and dispersed without aggregation, and the particle size (approximately 200 nm) was in accordance with the results shown in Table 3. In addition, the SEM and the particle size, zeta potential and polydispersity index were determined by drying samples. The results indicate that the lyophilization seems to basically have little influence on the shape and aggregation of the micelles.

### 3.4. Detection of Micelle Stability

**Figure 5:**
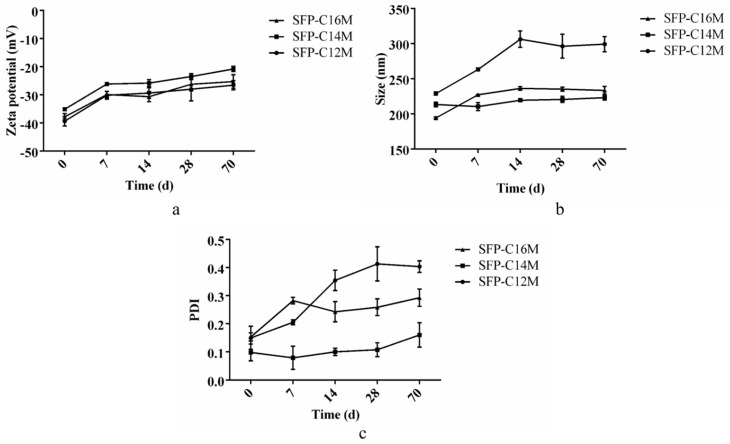
Determination of micelle stability. The results are the means ± SD (*n* = 3). (**a**) Changes in the zeta potential; (**b**) changes in the particle size; (**c**) changes in the PDI.

Particle size, zeta potential and PDI were measured on days 0, 7, 14, 28 and 70 to evaluate stability of the micelles. The results in Figure 5 showed that the zeta potential variation of the three micelles was similar. However, the particle size of SFP-C12M increased significantly from 229 nm to 305 nm from day 0 to day 14, while the sizes of the other two micelles did not significantly change from day 0 to day 70. Additionally, the PDI of SFP-C12M also apparently increased over time, reaching 0.413 on the 28th day, indicating that the micelles were heterogeneous. The PDI of SFP-C16M increased from 0.153 to 0.283 on the 7th day and remained stable. There was no obvious change in the PDI of SFP-C14M, which increased only from 0.108 to 0.160 from day 28 to day 70. The results above indicate that SFP-C14M and SFP-C16M were more stable than SFP-C12M. Therefore, it was supposed that a longer alkyl group led to stronger hydrophobicity and caused greater micelle stability.

### 3.5. Cytotoxic Activity

The toxicity of the SFP derivatives and nanomicelles to DF-1 cells is shown in Table 4. The results illustrate that the relative survival rate of DF-1 cells after treatment with SFP-C16 at a concentration of 1 mg/mL was higher than 95%. The safe concentrations of the other derivatives and micelles were 0.5 mg/mL. Therefore, the highest concentration of all samples in the following experiments was 0.5 mg/mL. Compared with our previous studies [11], the safe concentration of the SFP on DF-1 cells was 2 mg/mL. Therefore, the safe concentrations of the SFP derivatives and nanomicelles decreased and the SFP derivatives exhibited high cytotoxicity. Studies have shown that after modification with long alkyl chains, the cholesterol and lipid adsorption effects of citrus pectin, cellulose and chitosan are improved [48]. Gold nanoparticles modified with long alkyl chains inhibit HSV-1 (herpes simplex virus type 1), HSV-2 (herpes simplex virus type 2,), HPV-16 (human papillomavirus type 16) and RSV (respiratory syncytial virus). It was proven that the modified gold nanoparticles could destroy the virus lipid envelope [49]. Subsequently, we speculated that the derivatives and nanomicelles might interact or disrupt the biological membrane, resulting in increased toxicity.

### 3.6. The Anti-ALV-J Activity of SFP Nanomicelles In Vitro

**Figure 6:**
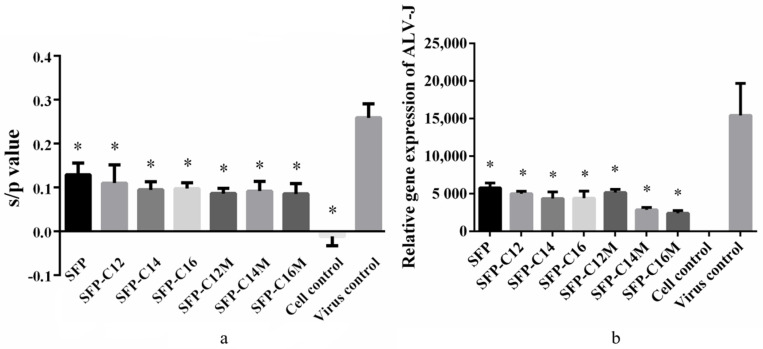
Antiviral effects of the SFP derivatives and nanomicelles. The results are the means + SD (*n* = 3); * *p* < 0.05 compared with the virus control. (**a**) Relative ALV p27 antigen expression; (**b**) relative ALV-J gene expression.

To generally evaluate the anti-ALV-J activity of the micelles, the ALV-J p27 antigen was detected. The s/p value (the relative expression of the ALV p27 antigen) greater than 0.2 was recommended as a positive result. The results in Figure 6a show that the s/p value of the virus control group was 0.258; however, the values in the other groups were significantly lower than those of the virus control group. The s/p value of the SFP group was 0.130, which was higher than that of the derivative and micelle groups. However, the relative p27 expression levels between the derivative and micelle groups were similar. Remarkably, the SFP derivatives and nanomicelles had greater inhibitory effects on ALV-J than the SFP.

In order to further evaluate the antiviral effects, RT-qPCR was used to detect the relative expression of the ALV-J gene. As shown in Figure 6b, the results of the virus control group reached 15,417.52, and those of the SFP group reached only 5780.108. Moreover, the results of SFP-C12, SFP-C14 and SFP-C16 were all lower than 5000. Additionally, the relative expression levels of the ALV-J gene in the SFP-C14M and SFP-C16M groups were 2858.225 and 2416.192, respectively, which were lower than those in the SFP-C14 (4368.369) and SFP-C16 (4399.448) groups, showing promising antiviral effects. The results above indicated that the SFP derivatives and nanomicelles could significantly suppress ALV-J and that SFP-C16M exerted the best inhibition. Katsuraya et al. [50,51,52] found that malto- and laminara-oligosaccharides modified with long-chain alkyl groups had a higher anti-HIV activity than unmodified oligosaccharides. Moreover, sulfated 3-O-octadecyl dextran also exerted better anti-HIV activity than standard dextran sulfates [53]. Bai et al. [54] prepared sulfated 1-(decadecyl-1,2,3-triazole)-1-deoxy-maltoheptaoside and determined the EC_50_ (concentration for 50% of the maximal effect) of the anti-HIV activity to be 0.03 μg/mL, which was significantly higher than that of maltoheptaoside sulfate (EC_50_ > 200 μg/mL). It was suggested that the long-chain alkyl group might penetrate or damage virus lipid membranes as surfactants, such as sodium dodecyl sulfate, do [54,55]. Thus, the introduction of a long-chain alkyl group played a pivotal role in the SFP derivatives, and the micelles exhibited enhanced anti-ALV-J activity.

### 3.7. Action Stage Assay Results

**Figure 7:**
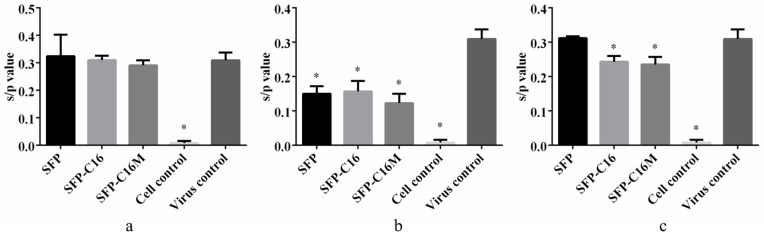
ALV-J p27 antigen expression after different administrations. The results are the means ± SD (*n* = 3); * *p* < 0.05 compared with the virus control. (**a**) BA (before adsorption) administration; (**b**) Ad (adsorption) administration; (**c**) AD (after adsorption) administration.

Based on the results above, SFP-C16 and SFP-C16M were screened to determine their action stage on ALV-J. Viral infection was artificially divided into three stages. The cells and the virus were treated with the indicated samples at different stages, and then the expression of the ALV-J p27 antigen was detected by ELISA. Studies have found that many polyanions, such as carrageenan [56,57,58], heparin [59], ulvan [60,61] and dextran sulfate [62], could inhibit virus adsorption onto host cells. Similar to our previous studies, the SFP played a role only in the virus adsorption stage (Figure 7b). Nevertheless, for the AD action stage (Figure 7c), the s/p values of SFP-C16 and SFP-C16M were 0.243 and 0.235, respectively, which were significantly lower than those of the virus control group. The results demonstrated that SFP-C16 and SFP-C16M also exhibited virucidal effects on ALV-J in the stage of virus replication. The SFP’s virucidal effects were not as strong as its adsorption prevention effects. This might be due to the low degree of substitution of the long-chain alkyl groups of the SFP derivatives.

### 3.8. GUV Exposure Experiment Results

Considering the results above, we speculated that the antiviral effects of the SFP nanomicelles on the AD stage were carried out through an interaction or direct destruction of the ALV-J phospholipid membrane. Therefore, we prepared GUVs to investigate whether the nanomicelles would damage them.

**Figure 8:**
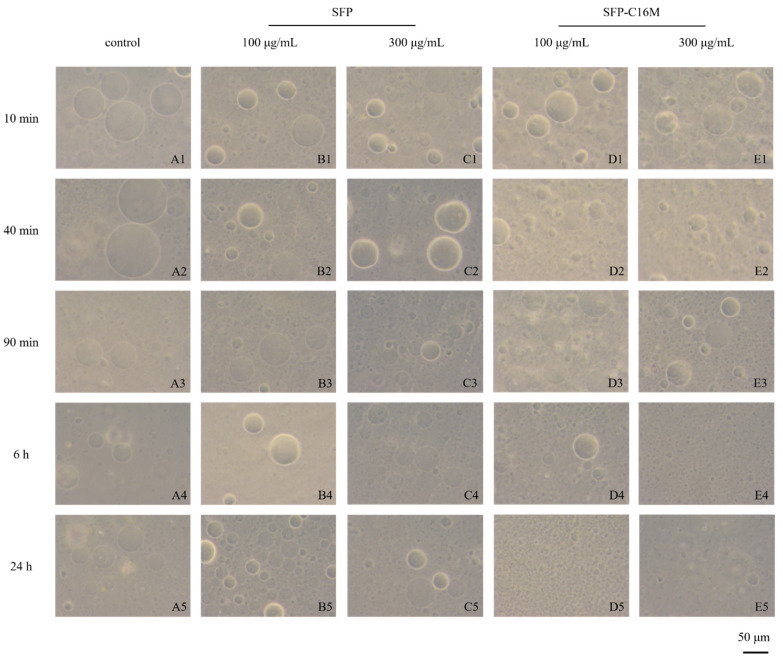
Morphology of GUVs^–^ after 10 min, 40 min, 90 min, 6 h and 24 h of exposure to the SFP and SFP-C16M at different concentrations (100 μg/mL and 300 μg/mL) (200×). (**A**): control group; (**B**): 100 μg/mL SFP group; (**C**): 300 μg/mL SFP group; (**D**): 100 μg/mL SFP-C16M group; (**E**): 300 μg/mL SFP-C16M group; scale bar = 50 μm.

During GUV preparation, charged lipids should be added to maintain the stability of GUVs. The cell membrane was negatively charged due to the presence of many polyanionic oligosaccharides, and negatively charged phospholipid molecules with hydrophobic heads were also in the outer layer. ALV-J virions contain 30–35% lipids, most of which are derived from host cell membranes. Consequently, it was appropriate to simulate the ALV-J virus envelope using negatively charged GUVs (GUVs^−^) rather than positively charged GUVs (GUVs^+^). The results in Figure 8 show the influence of SFP-C16M on GUV^–^ morphology. Spherical, uniform and dispersed GUVs^–^ were observed at all times in the blank control and SFP groups, which indicated that the SFP had no effect on GUVs^−^. However, the GUVs^–^ in the SFP-C16M group changed, gradually decreased and finally disappeared. For the 100 μg/mL SFP-C16M group, only a few large vesicles existed at 6 h (D4). At 24 h (D5), the large vesicles disappeared completely and were replaced by very small vesicles and black spots, which were lipid spheres formed by phospholipid fragments, indicating that the spherical structure of GUVs^−^ was completely destroyed. Figure 8E1–E5 show the morphological changes of GUVs^−^ from 10 min to 24 h after administration of 300 μg/mL SFP-C16M. At 40 min (E2), the shapes of some GUVs^−^ changed, and irregular spheres with crescent and half-crescent forms appeared. A few spherical vesicles could still be seen at 90 min (E3). However, at 6 h (E4), the large vesicles were completely destroyed and had disappeared from the field of vision, with only very small vesicles and fragments of phospholipids remaining. The influence of SFP-C16M on GUVs^+^ was also tested (Supplementary Materials, Figure S2). Because of the attraction between opposite charges, the SFP was adsorbed onto the GUVs^+^ and due to GUVs^+^ aggregation, a slight deformation of the GUVs^+^ was observed. However, the spherical and ellipsoidal GUVs^+^ were still clear at 24 h. Notably, the GUVs^+^ in the SFP-C16M group were severely destroyed at 90 min and totally fragmented at 24 h. Therefore, the SFP micelles could destroy the integrity of the GUVs. In addition, the higher the concentration of micelles and the longer the reaction time between the GUVs and the micelles, the more damaged the vesicles became. Bai et al. [54] found that the long-chain alkyl group of sulfated alkyl maltoheptaoside could penetrate the liposome bilayer, but the liposome was not destroyed. We speculated that this might be related to the degree of substitution of long-chain alkyl groups in the SFP derivatives. Each sulfated alkyl maltoheptaoside molecule carried only one alkyl group chain while each SFP nanomicelle derivative might carry several long alkyl chains, making SFP derivatives more permeable to the vesicle and able to cause more damage to the main structure of the vesicle, leading to the cleavage of the vesicles. However, the specific mechanism still needs further study. As described above, when SFP nanomicelles bind to the gp85 protein of ALV-J and inhibit ALV-J adsorption onto host cells, the long-chain alkyl group in the micelle might penetrate into the phospholipid membrane of ALV-J, causing damage to it, exposing the internal RNA of ALV-J and contributing to virion disintegration.

## 4. Conclusions

In conclusion, three SFP long-chain alkyl group derivatives were prepared, and the IR and NMR spectra showed that the syntheses were successful. Subsequently, the derivatives effectively self-assembled into nanomicelles by the dialysis method, and the physical and chemical properties of the SFP nanomicelles were characterized. The results revealed that the micelles were smooth, spherical, uniform and dispersed without aggregation, and the particle sizes were approximately 200 nm. In the antiviral experiment, SFP nanomicelles exhibited better antiviral effects than the SFP. In addition, compared to the SFP which only inhibits virus absorption onto host cells, SFP nanomicelles also exhibited antiviral effects in the virus replication stage. The results of the vesicle exposure experiments indicated that the new antiviral effect might be caused by damage to the ALV-J lipid membrane by the nanomicelles, resulting in inactivation of the virions. However, the specific antiviral mechanisms of the SFP and SFP nanomicelles still need to be further explored and verified.

## Figures and Tables

**Table 1:** Chemical composition of the SFP.

Sample	Total Sugars (%)	Sulfate (%)	Monosaccharide Composition (Molar Ratio)						
			Man	Rha	Glc A	Glc	Gal	Xyl	Fuc
SFP	54 ± 1	37 ± 1	0.34	0.15	0.18	0.01	0.72	0.19	1.00

Man, mannose; Rha, rhamnose; Glc A, glucuronic acid; Glc, glucose; Gal, galactose; Xyl, xylose; Fuc, fucose.

**Table 2:** Chemical properties of SFP long-chain alkyl group derivatives.

Sample	Yield (%)	Total Sugars (%)	Sulfate (%)
SFP		54.13	36.92
SFP-C12	9.57	52.03	34.64
SFP-C14	9.70	47.33	32.54
SFP-C16	8.09	47.29	30.96

**Table 3:** Properties of the SFP nanomicelles. The results are the means ± SD (*n* = 3), letters a, b, c in the same line represent a significant difference at *p* < 0.05.

Sample	Size (nm)	PDI	Zeta Potential (mV)	CMC (μg/mL)
SFP-C12M	229 ± 2 ^a^	0.15 ± 0.04 ^a^	−39.4 ± 1.7 ^a^	8.8
SFP-C14M	214 ± 3 ^b^	0.10 ± 0.03 ^a^	−35.1 ± 0.4 ^b^	5.0
SFP-C16M	194 ± 1 ^c^	0.15 ± 0.02 ^a^	−37.8 ± 1.1 ^ab^	12.1

**Table 4:** Relative survival rate of DF-1 cells.

Concentration (mg/mL)	2	1	0.5	0.25	0.125	0.0625	0.03125
SFP-C12	0.59	0.8	0.95	0.94	0.97	0.99	1.11
SFP-C14	0.73	0.85	0.96	1.11	1.04	1.02	1.04
SFP-C16	0.86	0.99	0.97	1.03	1.00	0.96	1.11
SFP-C12M	0.75	0.87	1.15	0.98	1.02	0.93	0.99
SFP-C14M	0.51	0.76	1.03	0.97	1.01	1.00	1.11
SFP-C16M	0.61	0.87	0.95	0.94	0.96	0.97	1.1

## Data Availability

The data used and/or analyzed during this study are available from the corresponding author upon reasonable request.

## Supplementary Materials

The following are available online. Figure S1: HPLC profile of the SFP, Figure S2: Morphology of GUVs after 10 min, 40 min, 90 min, 6 h and 24 h exposure to the SFP and SFP-C16M at different concentrations (100 μg/mL and 300 μg/mL) (200×).

## Sample Availability

Samples of the compounds are not available from the authors.
