# Supplementary material for: Preparation of New Sargassum fusiforme Polysaccharide Long-Chain Alkyl Group Nanomicelles and Their Antiviral Properties against ALV-J
Source: Molecules. 2021 May 28;26(11):3265. doi: 10.3390/molecules26113265 (PMC8199121; doi:10.3390/molecules26113265)
Supplement: Supplementary file 1 [file molecules-26-03265-s001.zip › molecules-1217200-supplementary.pdf]

Supplementary material

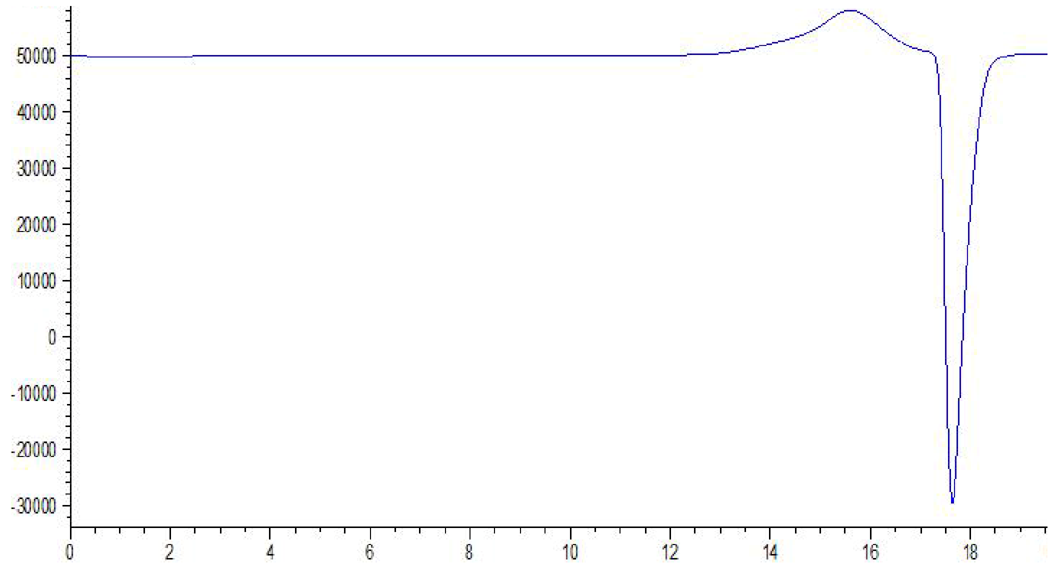

Figure S1. HPLC profile of SFP

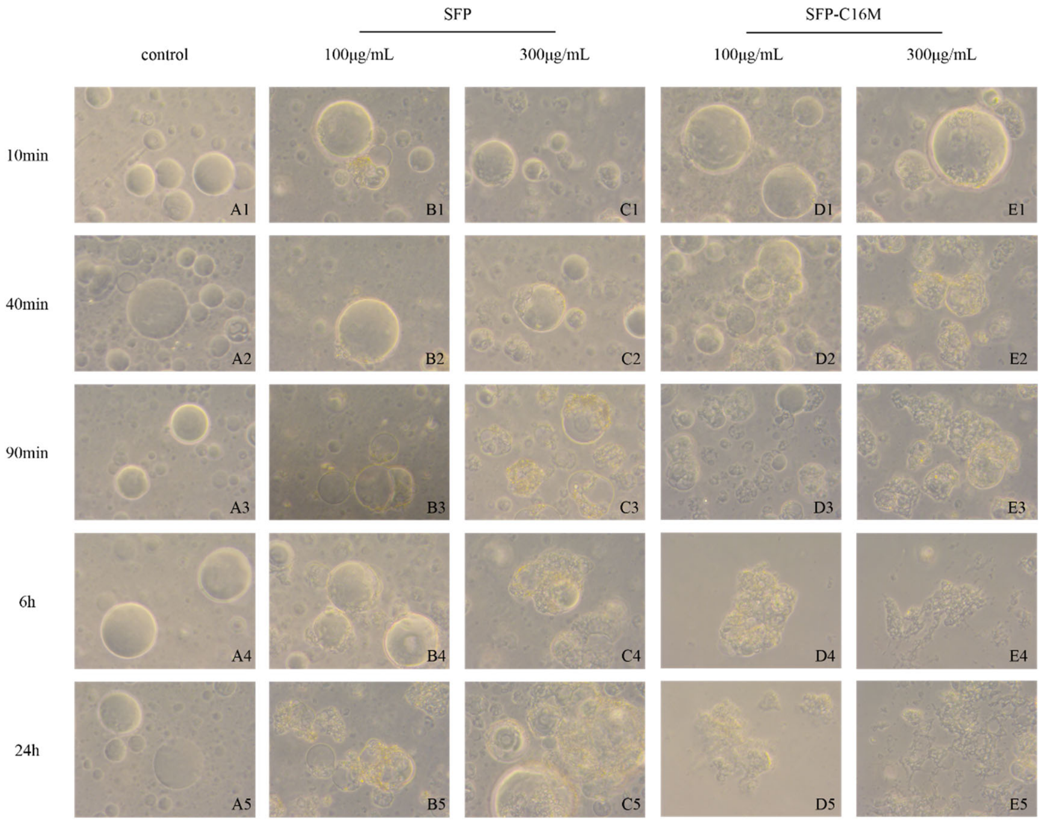

Figure S2. Morphology of GUVs- after 10min, 40min, 90min, 6h and 24h exposure in SFP and SFP-C16M of different concentration (100μg/mL and 300μg/mL) (200×).
